# Validity and Diagnostic Ability of Pancreatic Exocrine Insufficiency Questionnaire in Turkish Patients

**DOI:** 10.5152/tjg.2024.24061

**Published:** 2024-09-01

**Authors:** Dilek Oğuz, İsmail Hakkı Kalkan, Müjde Soytürk, Kadir Demir, Nevin Oruç, Göksel Bengi, Özlem Gül, Nalan Gülşen Ünal, Aslı Çiftibaşı Örmeci

**Affiliations:** 1 Department of Gastroenterology, University of Çanakkale On Sekiz Mart School of Medicine, Çanakkale, Türkiye; 2 Department of Gastroenterology, TOBB University of Economics and Technology School of Medicine, Ankara, Türkiye; 3 Department of Gastroenterology, University of Dokuz Eylül School of Medicine, İzmir, Türkiye; 4 Department of Gastroenterology, University of Istanbul School of Medicine, Istanbul, Türkiye; 5 Department of Gastroenterology, University of Ege School of Medicine, İzmir, Türkiye; 6 Department of Gastroenterology, University of Lokman Hekim School of Medicine, Ankara, Türkiye

**Keywords:** Diagnostic tools, pancreatic exocrine insufficiency, patient-reported outcomes, questionnaire validation, Turkish population

## Abstract

**Background/Aims:**

Pancreatic exocrine insufficiency (PEI) is a prevalent disease that is often underdiagnosed and undertreated, leading to resulting in diminished health-related quality of life. The PEI questionnaire (PEI-Q), a patient-reported outcome questionnaire developed to diagnose and evaluate PEI, is available only in English. The study aimed to provide a Turkish translation of PEI-Q and validate its reliability and diagnostic performance in a Turkish-speaking population with PEI.

**Materials and Methods:**

This study included 161 participants: 98 patients with PEI and 63 healthy controls. Participants underwent the PEI-Q test, and the results were statistically analyzed for reliability and validity. The diagnostic value of PEI-Q was determined using receiver operating characteristic (ROC) curves. Cronbach’s alpha was used to assess internal consistency, while exploratory factor analysis was performed to determine construct validity and reveal the factor structure.

**Results:**

: The mean age of participants was 45.0 years, and 60.2% were male. Participants with PEI were significantly older than those without. Scores for abdominal, bowel movement, and total symptoms were significantly higher in patients with PEI than in controls. ROC analysis revealed good diagnostic value for PEI-Q, with areas under the curve ranging from 0.798 to 0.851 for different symptom scores. Cronbach’s alpha coefficients were above 0.70, indicating good internal consistency, and exploratory factor analysis supported a 4-factor structure, accounting for 68.9% of the total variance.

**Conclusion:**

: The Turkish version of the PEI-Q is a reliable, easy-to-use, and valid screening tool for diagnosing PEI. It consistently assesses symptoms and quality of life in patients with PEI, helping to inform diagnosis and treatment.

**Main Points**

- Pancreatic exocrine insufficiency is often underdiagnosed and undertreated, affecting the quality of life for patients. Therefore, having a reliable diagnostic tool like pancreatic exocrine insufficiency questionnaire (PEI-Q) is of utmost importance.
- We found that scores for abdominal symptoms, bowel movements, and total symptoms were significantly higher in participants with pancreatic exocrine insufficiency (PEI), suggesting the questionnaire’s ability to effectively discriminate between patients with and without PEI based on symptomatology.
- This study concludes that the Turkish version of the PEI-Q is a valid and reliable tool for diagnosing PEI, providing consistent assessments for symptoms and quality of life.
- PEI-Q is the first tool specifically designed for individuals with PEI. The Turkish version’s reliability and validity make it a useful clinical instrument for better diagnosing and managing PEI in Türkiye.

## Introduction

Pancreatic exocrine insufficiency (PEI) is a medical condition characterized by the inadequate synthesis, activation, or early degradation of pancreatic digestive enzymes, leading to problems with digestion and absorption of nutrients.^1,2^ Pancreatic exocrine insufficiency can be stratified as primary or secondary. Primary PEI arises from the lack of exocrine pancreatic tissue, commonly seen in conditions like chronic pancreatitis (CP) and cystic fibrosis (CF), which are the most prevalent causes of PEI in adults and children/adolescents, respectively. Disturbances in pancreatic innervation can also lead to primary PEI. Secondary PEI occurs when pancreatic enzymes are secreted but cannot function effectively due to anatomical modifications such as gastrointestinal surgery, including partial or total resection of the pancreas, or improper activation or inactivation of pancreatic enzymes.^1–5^

Accurate and early detection of PEI in clinical practice is critical since it can lead to complications such as malabsorption, nutritional deficiencies, impaired growth, increased infection rates, cardiovascular events, a decrease in quality of life due to abdominal discomfort/distension, steatorrhea, and diarrhea.^3^ However, precise diagnosis in clinical practice is challenging due to the absence of a gold standard test for PEI. At present, diagnosis of PEI relies on a combination of symptoms, nutritional markers, and the limited availability of non-specific, non-invasive pancreatic function tests within the appropriate clinical context.^6–8^

The foundation of PEI treatment includes Pancreatic enzyme replacement therapy (PERT), smoking cessation, abstention from alcohol consumption, dietary counseling, and regular follow-up to optimize treatment outcomes.^9^ The goals of PERT are to achieve an adequate enzyme level for the proper digestion and absorption of fats and fat-soluble vitamins, thereby relieving symptoms related to maldigestion and normalizing the nutritional status of patients with PEI.^10^ However, a significant number of patients remain undiagnosed or receive inadequate treatment, resulting in the manifestation of notable PEI-related symptoms, a decline in health-related quality of life (HRQoL), and an increase in morbidity and mortality rates, particularly among patients with CP.^11–13^

There is no universally accepted diagnostic test for PEI or treatment response evaluation. Given the subjective nature of PEI symptoms, patient self-reporting offers the most reliable assessment. Therefore, implementing a patient-reported outcome (PRO) measure, such as a questionnaire, could prove invaluable in screening individuals at outpatient clinics with symptoms suggestive of PEI. Moreover, a standardized assessment could aid in guiding treatment decisions, monitoring patient symptoms, and enhancing communication between patients and healthcare providers, particularly for individuals who have already been diagnosed with PEI.

The first specialized PRO instrument for PEI, the PEI questionnaire (PEI-Q), was introduced by Johnson et al. in 2017.^14^ Its development involved extensive qualitative research with PEI patients and expert clinical input. A comprehensive psychometric evaluation of the instrument in 2019 further refined it.^15^

In this study, we aimed to measure the validity and reliability of the Turkish version of the PEI-Q, the first PRO questionnaire specifically for PEI.

## Methods

### Study Design, Setting, Location, and Date

This study was a cross-sectional study with a validation design conducted at 4 different University Hospitals (Ege University Hospital, İstanbul University Hospital, and Kırıkkale University Hospital) from 3 different geographical regions of Türkiye between June 2019 and June 2021.

### Participants, Inclusion and Exclusion Criteria

All adult patients (age ≥18 years) presented to the outpatient clinics of the abovementioned University hospitals were eligible for study inclusion. Patients diagnosed with PEI, including those with CP, CF, and diabetes mellitus (DM), were recruited during their regular visits to outpatient clinics. In order to be eligible for participation, patients had to have a confirmed diagnosis of PEI by a healthcare professional, with fecal elastase levels below 200 pg/mL and a diagnosis of either CF or CP. The fecal elastase test was used for diagnosing PEI in patients other than those with CP. Patients with PEI who had a history of other gastrointestinal conditions or had undergone gastrointestinal surgery were excluded from the study. To establish a comparison group, healthy individuals from the same outpatient clinics were included as control subjects. Controls with abdominal complaints or bloating were excluded. The final study population included 161 participants: 98 PEI patients and 63 healthy controls.

### Pancreatic Exocrine Insufficiency Questionnaire

The PEI-Q is a comprehensive instrument encompassing 26 items distributed across 10 domains, aiming to evaluate the symptoms and impacts of PEI within the preceding 7-day period. This questionnaire is divided into 2 sections. The first section comprises 17 items that assess symptoms such as pain, bloating, irregular bowel movements, nausea/vomiting, and eating habits. The second section includes 9 items that examine the impact of PEI on various aspects of HRQoL, including daily activities, emotional well-being, diet, social functioning, and sleep. Most items utilize a 5-point Likert scale accompanied by verbal descriptors. Additionally, one specific item offers response options ranging from “less than one per day” to “more than four per day,” while another item involves a 9-segment abdominal diagram. Higher scores on the questionnaire indicate more severe symptoms and a more significant impact on HRQoL.

### Turkish Translation of Pancreatic Exocrine Insufficiency Questionnaire 

#### Forward Translation:

Two bilingual translators independently executed the initial translation process from English to Turkish. The first translator, equipped with an understanding of the concepts inherent to the questionnaire, delivered a translation that closely matched the original instrument. Conversely, the second translator, unaware of the questionnaire’s objectives, offered an alternative translation. The original translators deliberated upon and reconciled any differences between the 2 translations.

#### Backward Translation:

The initially translated questionnaire was independently was back-translated to English to verify the translation’s accuracy. This strategy aimed to circumvent potential bias; the backward translators were not informed about the distinct concepts encapsulated in the questionnaire. Any misinterpretations or ambiguities in wording were identified and rectified throughout the back-translation process.

### Expert Committee Review

An expert committee was formed to finalize the translation. This committee systematically examined all iterations of the translated questionnaire to ensure that the final Turkish version mirrored the original in terms of semantic, idiomatic, experiential, and conceptual equivalence. After the committee’s approval, the finalized version of the translated questionnaire was subjected to a pilot test with a representative sample of the intended respondents. Upon completing the questionnaire, these respondents were invited to articulate their understanding of each questionnaire item and the corresponding response. Considering the feedback obtained, the expert committee reached a consensus on all items, resulting in the final iteration of the translated questionnaire (Appendix 1). The committee decided that no cultural adjustments or adaptations were necessary.

### Statistical Analysis

All statistical analyses were performed using Statistical Package for the Social Sciences (SPSS) for Windows version 17.0 (SPSS Inc., Chicago, Ill, USA). Data were presented as means and standard deviation (SD) or medians and interquartile ranges (IQR) for continuous variables and as counts and frequencies for categorical variables. The normality of continuous variables was checked using Kolmogorov–Smirnov and Shapiro–Wilk tests. Differences between 2 groups for continuous variables were analyzed using the Student’s *t*-test or Mann–Whitney *U*-test, depending on the normality of distribution. Categorical variables were compared using the chi-square test or Fisher’s exact test, as appropriate. In this study, the accepted type 1 error was 5%.

### Validation Tests

Internal consistency reliability of the items within the same domain was examined using Cronbach’s alpha coefficient. Cronbach’s alpha values greater than 0.7 were considered good internal consistency of the items within the measure, and an alpha value greater than 0.8 was considered excellent.

Inter-item correlations were evaluated to assess the degree of correlation between each item within the domains of the measure. Each item was crucial to contribute to the overall scale and did not merely correlate highly with 1 or 2 items. Correlations were analyzed using Pearson’s correlation coefficient for normally distributed variables and Spearman’s rank correlation for variables not normally distributed. Inter-item correlations were deemed acceptable if they fell within the range of 0.2 to 0.7. Correlations above 0.7 might suggest item redundancy, whereas correlations below 0.2 might suggest that an item does not belong to the measure.

An exploratory factor analysis (EFA) was conducted to determine the construct validity of the PEI-Q and to identify potential factors inherent in the data. As it was hypothesized that there was a relationship between the principal components method, the most frequently and quickly used method in practice, and the factors, the direct oblimin method was utilized. Kaiser−Meyer−Olkin (KMO) measure of sampling adequacy and Bartlett’s test of sphericity were performed. Kaiser−Meyer−Olkin values above 0.6 and a significant Bartlett’s test (*P* < .05) were considered appropriate for factor analysis. A KMO test value of 0.823 and a significant Bartlett’s test confirmed that the sample size was sufficient for EFA.

The extraction method used was principal axis factoring. To help determine the number of factors to retain, we used the Kaiser criterion (eigenvalues greater than 1), the scree plot, and the interpretability of the factors. After extracting the factors, a Varimax rotation was performed to achieve a more straightforward structure with greater interpretability, allowing each item to load on one factor.

Factor loadings of 0.4 or above were considered satisfactory for an item to be included in a factor. Cross-loading items (i.e., items that load on more than one factor) were assigned to the factor on which they had the highest loading. Factors were interpreted and named according to the nature of the items that loaded most highly on them.

The total variance explained by the factors, commonalities, and eigenvalues was reported. Communalities indicate the proportion of each variable’s variance that the factors can explain, while eigenvalues represent the amount of information captured by each factor.

### Receiver Operating Characteristic Curve Analysis

To evaluate the diagnostic ability of the PEI-Q questionnaire in detecting PEI, we conducted ROC curve analyses. We assessed and compared the sensitivity and specificity of the PEI-Q at different cutoff points and identified the optimal cut-off value that balances sensitivity and specificity. We reported the accuracy of PEI-Q using the area under the ROC curve (AUC). Confidence intervals (CIs) were calculated for the AUC to estimate the measure’s uncertainty. The optimal cutoff value was considered as the point on the ROC curve that maximized the Youden index (J), which is equal to sensitivity plus specificity minus one, calculated for each cutoff point. We plotted ROC curves to compare the diagnostic ability of the 3 domains of the PEI-Q test (abdominal symptom score, bowel movement symptom score, and total symptom score).

### Ethics Committee Approval

The study adhered to the principles outlined in the Good Clinical Practice Guidelines and the Declaration of Helsinki. Ethical approval was obtained from the Institutional Ethics Committee of Lokman Hekim University School of Medicine (approval number: 2023/60, date: April 5, 2023). Written informed consent was obtained from all participants prior to their involvement in the study. Potential participants were recruited for the study through invitations extended by their clinicians. They received an information letter and an Informed Consent Form (ICF) outlining the study’s purpose, procedures, and relevant details.

### Sample Size Estimation and Power Analysis

For validation studies, it is typically recommended to include at least 10 events (in this case, patients with PEI) per variable or domain being assessed. The PEI-Q tool includes 26 items, which are grouped into 10 domains. Consequently, our study aimed to recruit at least 100 PEI patients based on the guideline of 10 patients per domain. We aimed for a diseased-to-control ratio of 2 : 1. Post-hoc power analyses were conducted to verify the robustness of our study design. For the 3 major domains of the PEI-Q, our study demonstrated over 99% power to detect statistically significant differences in the mean scores.

## Results

### Study Population

The study comprised 161 participants, of which 98 were patients with PEI and 63 were healthy controls. The majority of PEI patients had CP (n = 70, 71.4%). Other conditions represented in the PEI patient group included type 2 DM (n = 19, 19.5%), type 1 DM (n = 7, 7.1%), and CF (n = 2, 2%).

### Demographic and Clinical Characteristics of Participants

The mean age of the participants was 45.0 years (SD: 13.5 years), and 60.2% (n = 97) were male. Participants with PEI were significantly older than those without PEI (*P* < .001). The demographic and clinical characteristics of the participants are presented in Table 1.

### Comparison of PEI-Q Results

The abdominal symptom score, bowel movement score, and total symptom score were significantly higher in patients with PEI than controls (Table 2).

### Diagnostic Value of Pancreatic Exocrine Insufficiency Questionnaire Test

**Figure 1.**
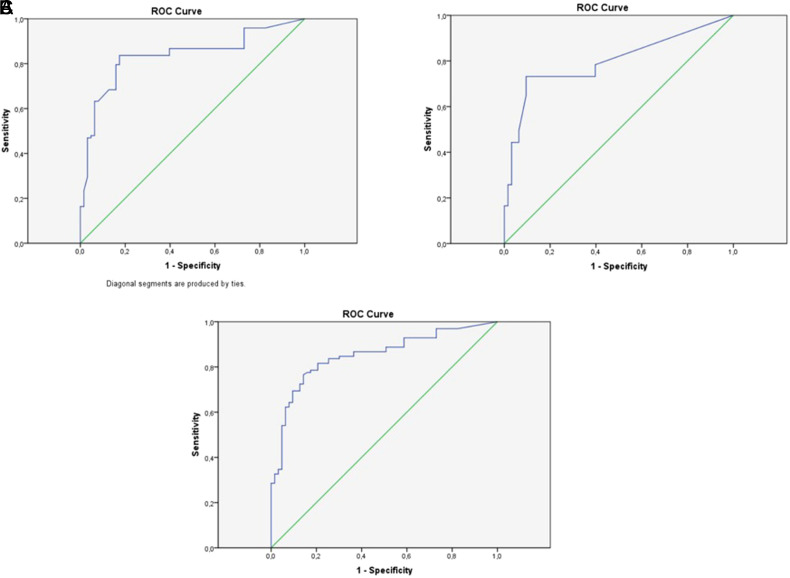
The ROC curves of the 3 domains of PEI-Q. (A) abdominal symptom score, (B) bowel movement symptom score, and (C) total symptom score.

The accuracy (area under the ROC curve, AUC) of the PEI-Q domains was calculated as follows and presented in Figure 1: 0.835 for the abdominal symptom score (sensitivity 79.6%, specificity 82.5% at the cutoff value of 0.71, *P *< .001), 0.798 for the bowel movement symptom score (sensitivity 73.2%, specificity 90.5% at the cutoff value of 0.33, *P *< .001), and 0.851 for the total symptom score (sensitivity 73.2%, specificity 90.5% at the cutoff value of 0.60, *P* < .001). These results indicate that PEI-Q has a good diagnostic value for PEI.

### Validation Tests for Pancreatic Exocrine Insufficiency Questionnaire 

#### Reliability Analyses:

The assessment of internal consistency, determined through Cronbach’s alpha coefficients, demonstrated good internal consistency with all alpha coefficients exceeding 0.70 (ranging from 0.718 to 0.880), as presented in Table 3. There were moderate correlations among the symptom items in section 1 of the PEI-Q, with only a few strong correlations exceeding 0.70. The majority of correlations fell within the range of 0.40 to 0.70. In comparison, the inter-item correlations were relatively weaker among the symptom items when compared to the impact items, as indicated in Table 4.

### Exploratory Factor Analysis

The Exploratory Factor Analysis revealed that the 15-item questionnaire comprised a 4-factor structure, accounting for 68.9% of the total variance, indicating the validity of the PEI-Q. Factor 1 accounted for 36.4% of the variance, factor 2 for 13.4%, factor 3 for 12.0%, and factor 4 for 6.9%. The minimum factor load was 0.429. As factor loads of 0.40 and above were considered ideal, the items significantly contributed to the factors. The distribution of items according to the factors and their respective factor loads is provided in Table 5.

## Discussion

The PEI-Q is a PRO questionnaire designed for individuals with PEI and represents the first tool. In this study, we aimed to validate the reliability and utility of the Turkish translation of PEI-Q and found that it is a valuable tool for diagnosing PEI and assessing its severity. Additionally, the PEI-Q demonstrates promising potential in monitoring treatment outcomes during patient follow-ups.^14,15^ This is the first Turkish validation study and the first non-English replication of the PEI-Q test, marking an important contribution to the literature.

Our results regarding internal consistency reliability and inter-item correlations suggest the PEI-Q has a robust internal structure, echoing the findings of the initial validation. An EFA further reinforced its construct validity and revealed a 4-factor structure. These 4 factors accounted for 68.9% of the total variance with factor loads of 0.49 (considered ideal if above 0.40), affirming the validity of the Turkish version of PEI-Q.

Pancreatic exocrine insufficiency significantly burdens individuals’ well-being and quality of life, even in its milder forms. The diagnostic complexity of PEI stems from the non-specific nature of mild to moderate disease presentations and the absence of a definitive diagnostic test. Traditional symptoms often manifest late in the disease progression, and the confirmation of PEI typically relies on the development of steatorrhea.^16^ However, it is crucial to note that no single symptom, including steatorrhea, can definitively establish or rule out the presence of PEI.

Integrating PROs into research studies has demonstrated substantial benefits in clinical practice. Protein-reported outcomes offer essential endpoints for evaluating the efficacy of treatments and assessing their potential adverse effects in clinical trials.^17,18^ By directly capturing patients’ experiences of symptoms and their impact on quality of life, PROs mitigate the potential bias introduced by observers in clinical trial assessments and enhance communication between patients and healthcare providers.^19,20^ Furthermore, the early inclusion of PROs in study design facilitates a more effective translation of research findings into clinical practice.^21–23^

Current guidelines for diagnosing and managing PEI in CP do not incorporate PROs.^24–27^ Instead, they rely on fecal elastase measurements, the only widely available test for PEI, and a pancreatic enzyme replacement therapy (PERT) trial period. However, the response to empirical PERT varies significantly in clinical trials. By taking into account additional symptoms, the PEI-Q enriches the benefits of a PRO in effectively monitoring PEI symptoms and capturing the comprehensive patient experience. This approach enhances the assessment of treatment response and facilitates the adjustment of PERT doses based on a standardized measure of patient-reported symptom severity.^15^

A notable strength of our study is the inclusion of patients predominantly with CF and CP, the primary etiologies of PEI, which aligns with the original PEI-Q. This strengthens our findings by providing evidence of content validity in diverse subgroups.

However, the study has some limitations. Data were collected in 4 different provinces (Ankara, Istanbul, Izmir, and Kirikkale), representing 3 of the 7 geographic regions in Türkiye. Nevertheless, it is essential to acknowledge that, like any self-reported questionnaire, the structure of the PEI-Q may be influenced by social desirability bias.

Pancreatic exocrine insufficiency is a prevalent disease that is often overlooked and inadequately managed,^11,12^ leading to a considerable negative impact on HRQoL and increased morbidity and mortality associated with malnutrition. To address this, our study presents a validated and user-friendly Turkish translation of the PEI-Q, serving as a valuable screening tool for diagnosing PEI. However, future research should explore the applicability and consistency of the PEI-Q in patients with PEI caused by factors other than CP or CF.

## Figures and Tables

**Table 1.** Comparison of Demographic and Clinical Characteristics of Participants

	**PEI Patients (n = 98)**	**Controls (n = 63)**	***P***
Age, mean (SD)	49.4 (14.3)	38.5 (9.0)	<.001
Male, n (%)	58 (59.2)	39 (61.9)	.7
Education level, n (%) * *University	33 (33.7)	51 (81.0)	<.001
Disease duration, n (%) * *<6 years >6 years * *N/A	56 (57.1) 35 (35.8) 7 (7.1)		

SD, standard deviation; N/A, not available.

**Table 2.** Comparison of the Mean PEI-Q Scores Between PEI Patients and Controls for all Domains

	**PEI-Q Test Scores, Mean (SD)**		
	**PEI Patients (n = 98)**	**Controls (n = 63) **	***P***
Abdominal symptom score	1.42 (0.84)	0.44 (0.46)	<.001
Bowel movement symptom score	0.87 (0.86)	0.15 (0.29)	<.001
Total symptom score	1.14 (0.74)	0.29 (0.33)	<.001

SD, standard deviation.

**Table 3.** Internal Consistency Analysis

	**Interclass Correlation Coefficient**	**95% CI**	***P***
Abdominal symptoms	0.832	0.784-0.871	<.001
Bowel movement symptoms	0.880	0.848-0.907	<.001
Impacts	0.793	0.718-0.853	<.001
Total symptom	0.718	0.418-0.842	<.001
Total summary	0.868	0.824-0.905	<.001

**Table 4.** Inter-item Correlation Analysis

	Abdominal Symptoms	Bowel Movement Symptoms	Impacts
A1	0.739	0.397	0.322
A2	0.693	0.416	0.236
A3	0.756	0.372	0.212
A4	0.712	0.310	0.155
A5	0.735	0.460	0.337
A6	0.621	0.488	0.196
A7	0.492	0.196	0.349
B8	0.448	0.829	0.320
B9	0.439	0.851	0.419
B10	0.374	0.754	0.280
B11	0.694	0.810	0.395
B12	0.237	0.640	0.253
B13	0.394	0.860	0.304
C14	0.106	0.170	0.554
C15	0.312	0.352	0.813
C16	0.284	0.292	0.698
C17	0.353	0.415	0.840
C18	0.359	0.328	0.822

**Table 5.** Exploratory Factor Analysis

Items	Factor 1	Factor 2	Factor 3	Factor 4
A1				–0.754
A2				–0.707
A3			0.720	
A4			0.936	
A5			0.791	
A6				–0.573
B8	0.823			
B9	0.796			
B10	0.794			
B13	0.896			
C14		0.429		
C15		0.804		
C16		0.705		
C17		0.825		
C18		0.870		
Eigenvalues	5.464	2.022	1.804	1.049
The variance explained	36.428	13.478	12.026	6.991
Total variance explained	68.923			
